# Structural Stabilization of Clinically Oriented Oligomeric Proteins During their Transit through Synthetic Secretory Amyloids

**DOI:** 10.1002/advs.202309427

**Published:** 2024-03-19

**Authors:** Julieta M. Sánchez, Hèctor López‐Laguna, Eloi Parladé, Angela Di Somma, Andrea L. Livieri, Patricia Álamo, Ramón Mangues, Ugutz Unzueta, Antonio Villaverde, Esther Vázquez

**Affiliations:** ^1^ Institut de Biotecnologia i de Biomedicina Universitat Autònoma de Barcelona Plaça Cívica s/n Bellaterra Barcelona 08193 Spain; ^2^ Departament de Genètica i de Microbiologia Universitat Autònoma de Barcelona Plaça Cívica s/n Bellaterra Barcelona 08193 Spain; ^3^ CIBER de Bioingeniería Biomateriales y Nanomedicina (CIBER‐BBN) Barcelona 08024 Spain; ^4^ Instituto de Investigaciones Biológicas y Tecnológicas (IIBYT) (CONICET‐Universidad Nacional de Córdoba) ICTA, FCEFyN, UNC Av. Velez Sarsfield 1611 Córdoba X5016GCA Argentina; ^5^ Department of Chemical Sciences University of Naples “Federico II” Vicinale Cupa Cintia 26 Naples 20126 Italy; ^6^ CEINGE Advanced Biotechnologies Via Gaetano Salvatore 486 Naples 80131 Italy; ^7^ Institut de Recerca Sant Pau (IR SANT PAU) Sant Quintí 77–79 Barcelona 08041 Spain; ^8^ Josep Carreras Leukaemia Research Institute Barcelona 08025 Spain

**Keywords:** cell‐targeting, drug delivery, microparticles, nanoparticles, recombinant proteins, secretory granules

## Abstract

Developing time‐sustained drug delivery systems is a main goal in innovative medicines. Inspired by the architecture of secretory granules from the mammalian endocrine system it has generated non‐toxic microscale amyloid materials through the coordination between divalent metals and poly‐histidine stretches. Like their natural counterparts that keep the functionalities of the assembled protein, those synthetic structures release biologically active proteins during a slow self‐disintegration process occurring in vitro and upon in vivo administration. Being these granules formed by a single pure protein species and therefore, chemically homogenous, they act as highly promising time‐sustained drug delivery systems. Despite their enormous clinical potential, the nature of the clustering process and the quality of the released protein have been so far neglected issues. By using diverse polypeptide species and their protein‐only oligomeric nanoscale versions as convenient models, a conformational rearrangement and a stabilization of the building blocks during their transit through the secretory granules, being the released material structurally distinguishable from the original source is proved here. This fact indicates a dynamic nature of secretory amyloids that act as conformational arrangers rather than as plain, inert protein‐recruiting/protein‐releasing granular depots.

## Graphical abstract

Polypeptides incorporated into artificial secretory amyloids, by the coordination between Zn^2+^ and histidine‐rich tails, undergo a conformation modification by which the protein gains structural stability, associated to a better secretory performance. These data offer clues for the mechanistic understanding of amyloidal depots with secretory properties and for the fabrication of high‐performance versions for clinical applications.

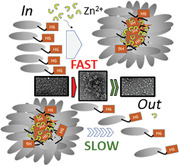

## 1 Introduction

Secretory granules from the mammalian endocrine system are functional, non‐toxic amyloids formed by the reversible coordination between histidine residues in peptidic hormones and cationic Zn.^[^ ^1^, ^2^, ^3^, ^4^ ^]^ The controlled clustering of His‐tagged functional proteins into insoluble granules results into artificial versions of such structures, that allow the time‐sustained release of proteins or protein drugs.^[^ ^5^, ^6^ ^]^ In contrast to other more complex slow drug delivery platforms, that require drug entrapping matrices,^[^ ^7^, ^8^, ^9^ ^]^ no holding matrices or porous containers are necessary here, since the building block protein is self‐retained in form of chemically homogeneous, self‐supported microscale particles. These protein clusters slowly disintegrate under physiological conditions, either in vitro or in vivo, into bioavailable polypeptides. Upon subcutaneous administration in mice models, such depots release functional proteins for at least 2 weeks being a convenient source of them under the drug delivery concept.^[^ ^6^ ^]^ Avoiding the potential toxicity of any molecular container and resulting from a very simple aggregation process, artificial secretory granules show promises in oncology and in regenerative medicine, among other clinically relevant fields.^[^ ^10^ ^]^ Being fabricated in vitro out of pure polypeptide species, the clustering and disintegration processes followed by the protein are irrespective of its source. Then, both chemical peptide synthesis but also conventional production of recombinant proteins in any category of cell factories are suited as a source of building blocks.

The disintegration of the protein material and the resulting protein leakage is assumed to be based on a natural chelation or dilution of the clustering metal and the consequent and progressive detachment of the building blocks, that once released become soluble and remain functional. However, the mechanics of protein clustering and release are rather neglected, a fact which precludes a fine tuning of artificial secretory granules for specific applications. Also, a clearer comprehension of the architecture and performance of artificial secretory granules based on ionic Zn would eventually allow a better control of the protein release rate through refined fabrication protocols. By taking as a model the protein‐only oligomeric nanoparticle T22‐GFP‐H6, of interest in clinical oncology,^[^ ^11^, ^12^ ^]^ and fully supported by additional, structurally unrelated polypeptides (β‐Gal‐H6, EPIX4‐RK‐GFP‐H6 and GFP‐H6), we have dissected here the disintegration event of secretory granules and the structural reorganization undergone by the released material throughout its clustering as protein microparticles. Interestingly, the obtained data reveal that the secretory granules are not inert depots to which the building blocks are merely attached and further detached. In contrast, the embedded protein undergoes conformational adaptations throughout their transit through the granules that result in the modification of its structure and oligomeric status. Then, the incoming (*IN*, never aggregated) and outcoming (*OUT*, released from aggregates) protein materials are structurally distinguishable. Since such conformational transition is modulated by the particular His‐coordinating agent used for the aggregation, this information opens a way for the refined and rational construction of protein‐based biomaterials that are unexpectedly dynamic regarding the folding and the supramolecular status of their components.

## 2 Results

**Figure 1:**
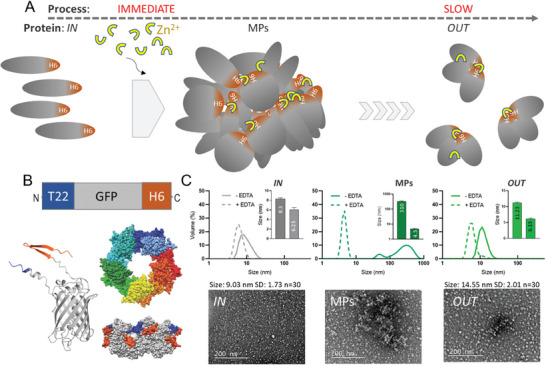
Protein transit though secretory granules. A) A generic model for the fabrication and performance of secretory granules. Homologous polypeptides (grey), tagged with H6 (orange, *IN* protein), are immediately clustered together as insoluble, microscale particles (MPs) by the addition (fine arrow) of cationic Zn (yellow). Protein clustering takes place in a very fast aggregation process, resulting in its immediate precipitation upon the addition of the metal (single big grey arrow). The formed MPs slowly release those building block polypeptides (*OUT* protein) during a prolonged period of time, in a slow leakage process (multiple small grey arrows). Such release takes place under physiological conditions, both in vitro and in vivo. B) Modular architecture of T22‐GFP‐H6. T22 is an efficient ligand of the cancer cell marker CXCR4, whose presence does not disturb the fluorescence of the His‐tagged GFP. At the bottom, left, an alphafold‐based model of the T22‐GFP‐H6 monomer. At right, a putative but validated 3D surface representation of self‐assembled T22‐GFP‐H6 nanoparticles in a top view (modelled with HADDOCK, each monomer differently coloured). A side view of this model is also displayed in which T22 is shown in orange and H6 in blue. Reproduced from ^[^ ^17^ ^]^ with permission from Wiley. C) Dynamic light scattering (DLS) analyses of T22‐GFP‐H6 *IN* nanoparticles, the resulting secretory granules (*MPs*) and the final, secreted *OUT* nanoparticles. DLS plots of EDTA‐treated materials (dotted lines) are also shown to test the reversibility in the assembly of all (*IN*, MPs and *OUT*) materials (continuous lines). The basic, common building block shows a hydrodynamic size between 4 and 6 nm, compatible with the GFP monomer or dimer. Broad field TEM images of *IN* and *OUT* protein samples are shown, indicating the average size of the particles in n = 30 counts. A representative image of MPs is also included, that highlights the aggregate nature of the protein in the precipitated state.

Poly‐histidine tagged proteins can be simply aggregated by the addition of a molar excess of cationic Zn, in a fast process that occurs immediately upon the addition of either this metal or other types of divalent cations (**Figure** **1**A). The resulting micron particles (MPs), with amyloidal architecture, spontaneously disintegrate under physiological conditions, both in vitro and in vivo, in a time‐prolonged process.^[^ ^6^ ^]^The clinical potential of such platform for long‐term protein release in the context of drug delivery is supported by the full functionality of the leaked protein. In fact, the whole concept mimics the performance of secretory granules (also functional amyloids) from the human endocrine system where peptide hormones are stored and further released.^[^ ^1^, ^2^, ^3^, ^4^, ^13^ ^]^ However, it has not been determined if the incoming building block protein (*IN*) is merely attached and detached by the incorporation and chelation of the linking metal, or if in contrast, it undergoes conformational conversions during its transit through the MPs. In other words, it is not known how much the *IN* and *OUT* (released) protein match from a structural point of view.

To approach this issue, we have selected the modular construct T22‐GFP‐H6 as a study model (Figure 1B). This modular fusion, containing the green fluorescent protein (GFP), was produced in bacteria and upon purification and it spontaneously assembles into homo‐oligomeric nanoparticles of ≈9 nm in size, assisted by divalent cations (Figure 1B,C). This protein construct aggregates as insoluble MPs of between 300 and 400 nm upon addition of a molar excess of cationic Zn (Figure 1C). After incubation in buffer, the electrophoretic mobility of the *OUT* material under non‐denaturing conditions was supportive of its oligomeric form (Figure S1A, Supporting Information), and such supramolecular organization was fully confirmed by size exclusion chromatography (Figure S1A, Supporting Information). When exploring the size of *IN* and *OUT* T22‐GFP‐H6 oligomers upon incubation of MPs in buffer, for 7 days at 37 °C, we observed a slight but clear size increase of a few nm (Figure 1C), from both transmission electron microscopy (TEM)‐ and DLS‐based observations (small differences in the absolute size values depend on the used method). This result, although preliminary, could indicate a possible structural modification of the material during its occurrence in the granules. The disassembly of all these types of materials by the chelating agent EDTA (Figure 1C) confirms, apart from the clustering capabilities of ionic Zn, the reversible nature of the supramolecular entities and the homogenous hydrodynamic size of the common building block, ≈4–6 nm (compatible with the dimensions of the T22‐GFP‐H6 monomer or dimer).

**Figure 2:**
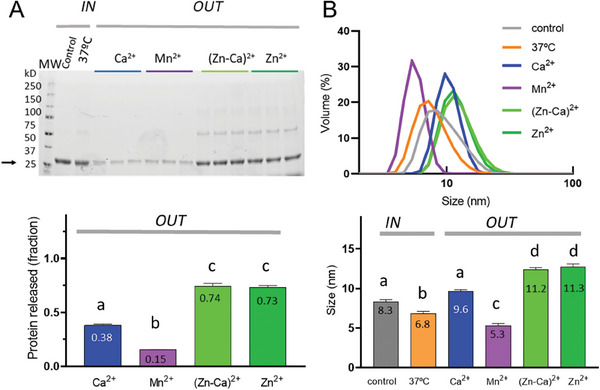
Protein release from secretory granules. A) Electrophoretic analysis of *OUT* T22‐GFP‐H6, released from MPs formed by either Zn^2+^ or alternative clustering cations. Samples were taken upon incubation in buffer, for 7 days at 37 °C, of freshly prepared granules, and processed in triplicate. *IN* protein either kept at 4 °C (Control) or stored for 7 days at 37 °C are also shown as references. The fraction of protein released is shown in the bottom panel. B) DLS plots of the *IN* and *OUT* materials shown in panel A. At the bottom, analyses of pairwise compared material sizes. In each plot, different letters over the bars means statistically significant difference (*p*<0.001) and equal letters mean no differences. Letter selection is irrelevant.

To analyses further the possibility of structural differences between the original *IN* and the secreted *OUT* materials, we determined the nature of protein release from MPs formed by Zn‐mediated clustering or by means of the coordination with other divalent cations. The amount of *OUT* protein was clearly distinguishable depending on the clustering agent. MPs formed by Zn (alone or in combination) were those showing a higher fraction of released protein after 7 days (**Figure** **2**A; Figure S1B, Supporting Information). The fine size analysis of the *OUT* material comparing to the *IN* version confirmed a significantly larger size in the nanoparticles released from Zn‐based MPs, no significant variation in the case of the Ca‐based material and a size reduction in Mn‐based materials (Figure 2B). The *IN* protein incubated at 37 °C for 7 days also showed a size reduction below 7 nm, compatible with its disassembly into protomers, either monomers or dimers (Figure 1C). This possibility was confirmed for both the *IN* protein at 37 °C and the Mn‐based granules by non‐denaturing electrophoresis (Figure S2A, Supporting Information), suggestive of dimers as building blocks or protomers.

**Figure 3:**
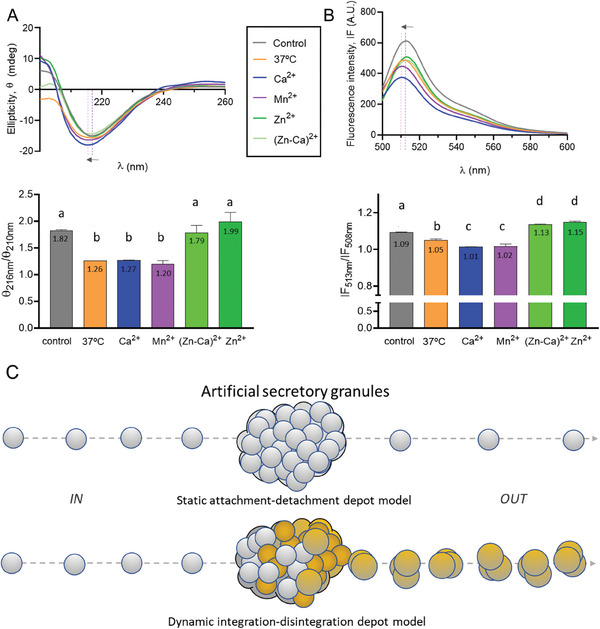
Comparative structure of *IN* and *OUT* materials. A) Ellipticity θ (in mdeg) versus wavelength (in nm) and θ_216_/θ_210_ ratio of *IN* and *OUT* T22‐GFP‐H6. B) Fluorescence emission and tertiary structure parameter (TSP = IF_513_/IF_508_ ratio) of *IN* and *OUT* T22‐GFP‐H6. Experimental conditions were as in Figure 2. Dashed lines and the arrow indicate changes toward lower wavelengths of the minimum ellipticity (ME) and maximum fluorescence intensity (MF) of the spectrum. Different letters over the bars mean statistically significant difference (*p*<0.001) and equal letter mean no differences. Letter selection is irrelevant. C) In a static attachment‐detachment depot model of secretory amyloids, building blocks are merely clustered and released according to the availability of cationic Zn. *IN* and *OUT* materials are conformational analogues. In a dynamic integration‐disintegration depot version, the building blocks become structural part of the granules and they undergo conformational adaptations during the building of the supramolecular material. The *OUT* material is structurally distinguishable from the *IN* material as it exhibits an oligomeric organization that also involves the tertiary structure of the protein. In the dynamic model, the disintegration process releases material more efficiently than in a mere passive detachment.

The observed Zn‐mediated modification of the nanoparticle size, when comparing *IN* and *OUT* materials, is probably based on conformational alterations in the building blocks during the transit through MPs. To explore this hypothesis, *IN* and *OUT* proteins were structurally compared. In general terms, all T22‐GFP‐H6 samples showed a dominant β‐sheet conformation. This is so as their circular dichroism spectra present a minimum ≈216 nm (**Figure** **3**A) ^[^ ^14^ ^]^ and a very close maximal fluorescence intensity (MF) wavelength (Figure 3B), that could be related to similar protein conformation. Under a deeper analysis, it was noted that the minimal ellipticity value (ME) (Figure 3A) and the MF wavelengths (Figure 3B) of *IN* T22‐GFP‐H6 moved to lower values when the protein was incubated at 37 °C for 7 days (see the arrow in the Figure 3A). However, the *OUT* protein versions released from Zn‐containing MPs (but not from alternative, Zn‐lacking MPs) showed unchanged CD spectral signals, although all MP types had been submitted to the same incubation conditions. These spectral behaviors indicate that the secondary structure (Figure 3A) of *OUT* T22‐GFP‐H6 from Zn‐based MPs remained the same as the control *IN* sample (kept at 4 °C) and different from the protein sample incubated at 37 °C. On the other side, the fluorescence signals that account for the tertiary structure of the protein showed that *OUT* proteins behave in a way different than that of the control at 4 °C and that of the *IN* sample kept at 37 °C (Figure 3B; Figure S2B, Supporting Information). It is noteworthy that the tertiary structure of *OUT* proteins leaked from Zn‐based MPs is different from the rest of *OUT* proteins, a fact that could be related to the observed size increase (Figure 1C and Figure 2B). In summary, there is conformational stabilization of the secondary structure in the building block protein T22‐GFP‐H6 by its transit through Zn‐based MPs (Figure 3C), involving a rearrangement of the oligomeric state resulting in an increase of the oligomer size (Figure 1C and Figure 2B).

**Figure 4:**
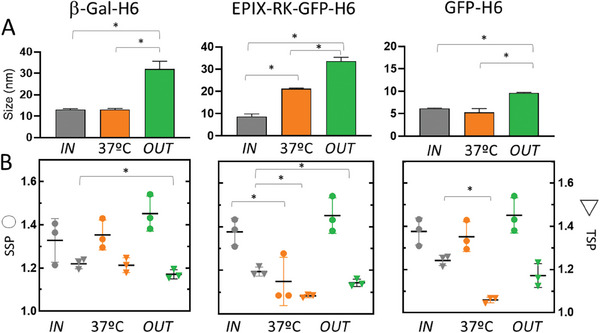
Protein‐independent modification of size and structure from *IN* to *OUT* materials. A) Hydrodynamic size of *IN* (grey) and *OUT* (green) versions of β‐Gal‐H6, EPIX‐RK‐GFP‐H6, and GFP‐H6, determined by DLS. Data from a control *IN* protein incubated at 37 °C (orange) is also shown as a reference. B) Secondary structure parameter (SSP) represented by circles calculated as θ_216_/θ_210_ ratio from CD spectra of the *IN*, *OUT* and 37 °C‐stored control proteins. Tertiary structure parameters (TSP) represented by triangles calculated as IF340/IF360 (β‐Gal‐H6) and IF513/IF508 (EPIX‐RK‐GFP‐H6 and GFP‐H6) ratios from the protein fluorescence emission. Asterisks indicate significant differences (*p*<0.015).

To discard that the observed structural arrangement could be restricted to a particular amino acid sequence, the *IN/OUT* comparative study was extended to a set of other oligomeric proteins. These include the large, natural tetrameric β‐galactosidase (in its His‐tagged form β‐Gal‐H6) and the tumor‐targeted modular construct EPIX4‐RK‐GFP‐H6.^[^ ^15^ ^]^ GFP‐H6, that under physiological conditions does not oligomerize,^[^ ^16^ ^]^ was also included. The expected size increase through MPs was again observed when comparing these additional *IN* and *OUT* versions (**Figure** **4**A). These data confirmed that the previous observations in this regard (Figure 1 and Figure 2), were not associated to the particular polypeptide T22‐GFP‐H6. Interestingly, GFP‐H6, that in its *IN* version sized ≈5 nm, was released from MPs as an oligomer of ≈10 nm (Figure 4A), thus proving a supramolecular adaptation during its transit. While in general terms, the secondary structure remained unchanged when comparing the *IN* and *OUT* versions (note the SSP in Figures 3, 4 and the secondary structure rate in Table S1 (Supporting Information), a modification of the tertiary structure of all proteins indeed occurred (Figure 3B, and TSP in the Figure 4B), probably linked to a more efficient protein leakage from the aggregates (Figure 2A). The modified spectral behavior of β‐Gal‐H6 upon its transit by MPs (Figure S3, Supporting Information) confirmed again the structural adaptation for a highly complex protein fully unrelated with the rest of the protein set.

**Figure 5:**
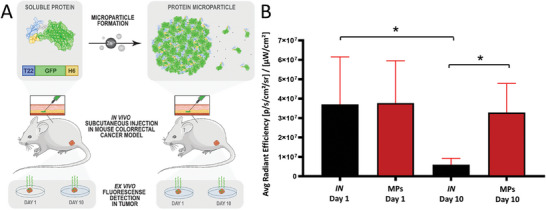
Comparative tumour accumulation of T22‐GFP‐H6 administered as *IN* soluble protein or Zn‐promoted MPs. A) Diagram illustrating the protein administration protocol in a murine model of CXCR4^+^ colorectal cancer. The scheme features protein nature, injection site and designated ex vivo tumour observation days. B) Accumulation of GFP fluorescence in tumour at days 1 and 10 after the administration of 1 mg of T22‐GFP‐H6, in the two alternative formats, namely *IN* or MPs. Asterisks indicate significant differences (*p*<0.05). Mann–Whitney test was applied for comparisons.

The secretory granules in the mammalian endocrine system, that store peptide and protein hormones, show a Zn‐sustained amyloidal architecture that allows the release of the building blocks for secretion.^[^ ^3^, ^4^ ^]^ To evaluate if the protein leakage observed here from the in vitro generated MPs might be comparable to the endocrine function we first determined the potential amyloidal organization of MPs. As observed using attenuated total reflectance with Fourier transform infrared spectroscopy (ATR‐FTIR) analysis (Figure S4, Supporting Information), there was a high amyloidal content in the four types of studied MPs, which was confirmed, in the case of β‐Gal‐H6, by Thioflavin T staining (Figure S4A, Supporting Information). Indeed, the amyloid content was important and not mere anecdotic, as it ranged from 31% to 44% (Figure S4B, Supporting Information). Such amyloid nature of MPs and their protein‐leaking features (Figure 1 and Figure 2) prompts the consideration, tailoring and envisaging of MPs as dynamic depots of protein oligomers for a clinically oriented, endocrine‐like, time‐sustained supply of functional proteins. Based on such concept, MPs, upon convenient administration, should result in steadier levels of circulating protein than when such protein is administered in its soluble version, representing a convenient drug release system with intriguing applicability. To test this possibility, we subcutaneously administered the same amount of T22‐GFP‐H6, in both soluble (*IN*) and MP versions, in a mouse model of CXCR4^+^ colorectal cancer. In vivo, the conventional soluble version (*IN*) of T22‐GFP‐H6 transiently accumulates in tumour because of the solvent‐display of T22 on the oligomers,^[^ ^17^ ^]^ the highly selective CXCR4‐binding and cell penetrability shown by T22,^[^ ^11^ ^]^ and the stability of the oligomeric nanoparticles once systemically administered.^[^ ^18^ ^]^ The maximal amount of protein in tumour usually peaked within the first hours after injection.^[^ ^19^ ^]^ In this context, we were particularly interested in knowing if the MP protein version, used as granular depot, could render time‐prolonged permanence of the protein in tumour because of its time extended delivery from the material, compared with the single shoot and consequent up and down levels of the *IN* version. Indeed, at day 1 upon administration of 1 mg of protein in either version (**Figure** **5**A), the amount of protein in tumour, reached from the subcutis via bloodstream, was indistinguishable when comparing the alternative formulations. However, after 10 days, T22‐GFP‐H6 was significantly present in the tumor when administered as MPs compared to its delivery as *IN* protein (Figure 5B). Therefore, the T22‐GFP‐H6 MPs acted as dynamic depots of functional protein that was delivered in a time‐prolonged way, through a precise tumour targeting via T22‐CXCR4 interaction and in an endocrine‐like fashion. Importantly, the efficient targeting observed here fully supported the proper folding and functionality of the stored protein upon release.

## 3 Discussion

Protein aggregation is a common event in nature,^[^ ^20^, ^21^, ^22^ ^]^ and it is involved in many functionalities of healthy cells and organisms ^[^ ^23^, ^24^, ^25^, ^26^, ^27^ ^]^ as well as in severe pathologies such as Parkinson and Alzheimer disease,^[^ ^28^, ^29^ ^]^ ageing ^[^ ^30^, ^31^ ^]^ and prion diseases.^[^ ^32^ ^]^ Among the wide spectrum of protein aggregates, a certain category of amyloidal structures, the so‐called non‐toxic functional amyloids, are involved in many organic functions including virulence in pathogenic bacteria,^[^ ^33^ ^]^ prevention of protein degradation through inclusion bodies (as a quality control mechanism) in bacteria ^[^ ^34^ ^]^ and peptide hormone storage and secretion in the mammalian endocrine system through secretory granules.^[^ ^4^, ^13^ ^]^ Both bacterial inclusion bodies and mammalian secretory granules act in fact as dynamic protein depots from which functional proteins are released in nature or in artificial settings.^[^ ^1^, ^2^, ^3^, ^4^, ^12^, ^35^ ^]^

From a thermodynamic point of view, amyloids represent the lowest free energy state achievable by proteins.^[^ ^2^ ^]^ However, data accumulated in the last years demonstrate the reversibility in the amyloid formation and the consequent protein releasability from amyloidal aggregates.^[^ ^36^, ^37^ ^]^ Then, pathogenic as well as functional amyloidal granules are in fact dynamic depots of either cytotoxic or functional proteins, that in humans are mainly hormones.^[^ ^1^, ^2^ ^]^ The studies demonstrating the reversibility of amyloid formation have been mainly pushed by research in the hormone secretory system in humans, mainly focusing on the pituitary gland.^[^ ^1^, ^4^, ^38^ ^]^ Importantly, the generic occurrence in nature of amyloids as depots for protein hormones (somatostatin, growth hormone (GH), corticotropin‐releasing factor, urocortin, vasoactive intestinal peptide, sCalcitonin, sfUrotensin, glucagon‐like peptide, extendin 4, bombesin, β‐endorphin, gastrin‐releasing peptide, oxytocin, secretin and many others ^[^ ^2^ ^]^) straightforward implies the reversibility of amyloid formation and the capability of these structures to release monomers upon appropriate environmental conditions.

First, monomers and amyloidal oligomers occur as an unbalanced association‐dissociation equilibrium ^[^ ^37^, ^39^ ^]^ that can be displaced toward dissociation by several mechanisms progressively identified (but not completely understood as a whole). An increase in the conformational flexibility of a protein (for instance by S‐S cleavage) favors aggregation and impairs detachment of monomers from the core fibrillar structure.^[^ ^39^ ^]^ On the other hand, cationic Zn (as used here) initiates the oligomerization of histidine‐containing proteins (such as prolactin ^[^ ^40^ ^]^ and GH ^[^ ^3^ ^]^), while dilution (or chelation ^[^ ^3^, ^41^ ^]^) of this metal in the media promotes protein leakage from amyloids. Also, ionic cooper triggers disassembly of neurokinin B functional amyloids,^[^ ^42^ ^]^ probably by blocking the imidazole group from His_3_, a critical residue in the assembly (and disassembly) of this neuropeptide as secretory amyloids.^[^ ^42^, ^43^, ^44^ ^]^

How organic signals induce the disassembly of secretory amyloids for protein hormone release, upon functional needs, is not yet clear. An interesting attempt in this regard is that done regarding the secretion of GH from the pituitary gland,^[^ ^38^ ^]^ whose homeostasis might require dozens of kinases and phosphatases. The releasability of the hormone in response to resistance exercise stress may involve S‐S isoforms and Zn availability, in addition to a structural variability in the secretory granules within the pituitary somatotroph.^[^ ^38^ ^]^

Regarding this structural variability, it must be noted the case of other functional amyloidal depots found in recombinant bacteria, namely inclusion bodies.^[^ ^45^ ^]^ In them, protease resistant amyloid fibrils ^[^ ^46^ ^]^ generate stable nets in which variable proportions of quasi‐native polypeptides are embedded by stereospecific interactions.^[^ ^47^ ^]^ Such a fraction of detachable protein can be easily released upon subcutaneous administration in mice for functional activity,^[^ ^12^ ^]^ probably by dilution of the cationic Zn, known to stabilize the whole depot complex through His residues.^[^ ^48^ ^]^

Because of the clinical applicability of such a depot/secretion system, we have recently developed clinically oriented mimetic versions of amyloid‐based protein depots, based on tagging proteins of interest with the conventional hexahistidine (H6) segment, which show applicability as time‐prolonged drug delivery systems.^[^ ^5^, ^49^ ^]^ As in their natural counterparts,^[^ ^44^, ^48^, ^50^, ^51^, ^52^, ^53^, ^54^ ^]^ these materials organize as submicron/micron granules resulting from the Zn^2+^ (and alternative divalent cations) coordination with solvent‐exposed histidine residues. While Zn^2+^ can coordinate with residues other than His,^[^ ^55^ ^]^this amino acid, added to the media in molar excess and as a soluble form, disassembles H6‐tagged protein oligomers induced by Zn.^[^ ^56^ ^]^ This fact indicates a main role of the His residues displayed by the protein in the clustering process. Such Zn‐His coordination, reversible upon chelation of the metal,^[^ ^57^ ^]^ cross‐links polypeptides into insoluble clusters with amyloidal architecture,^[^ ^5^, ^6^ ^]^ and it offers a possibility for the generation of artificial protein materials with secretory properties.^[^ ^10^, ^58^ ^]^ In contrast to bacterial inclusion bodies, that despite their applied interest ^[^ ^59^, ^60^ ^]^ are intrinsically contaminated with bacterial molecules,^[^ ^61^, ^62^, ^63^, ^64^ ^]^ the artificial secretory granules are self‐contained and chemically homogeneous, and they might consequently face a regulatory‐compliant route to clinics when exploring their self‐delivery properties.

By using a protein oligomer usable as drug carrier in oncology as a model,^[^ ^11^ ^]^ we have demonstrated that artificial secretory granules are not inert depots to which proteins attach and detach according to the availability of ionic Zn, but dynamic structures in which polypeptides undergo conformational transitions (Figure 3A,B and Figure 4B). Such modifications, observable when Zn is the clustering agent (Figure 3A,B and Figure 4A), stabilize the oligomers and allow a more efficient release and therefore, a higher bioavailability of the protein under physiological conditions (Figure 2A). A slight size increase in the nanoparticle size (Figures 1, 2,4A) has revealed such adaptation and it is probably the result of adapted protein‐protein contacts. A structural expansion of the protein within the released oligomers was also demonstrated with *OUT* β‐Gal‐H6 (Figure S3, Supporting Information), as the Trp population sensed a higher hydrated environment. In contrast, ionic Mn, as a clustering agent, is unable to keep the oligomeric status of the protein during the tested 7‐days period (Figure 2B; Figure S2, Supporting Information).

Regarding the in situ conformational adaptation determined here with T22‐GFP‐H6 and other model proteins, we have observed a high amyloidal content in the different MPs (Figure S4, Supporting Information) similar to the level found in bacterial inclusion bodies.^[^ ^5^ ^]^ Both protein particles behave as structurally changing entities in which the embedded polypeptides undergo dynamic modifications. In inclusion bodies, these changes, inside the cell, include aggregation, solubilization, refolding, functional activation and proteolytic digestion.^[^ ^61^, ^65^ ^]^ This is because the integration of these depots into the quality control system that involves the intense activity of chaperones and proteases, both in the cytoplasm but also inside or at the surface of the protein aggregates.^[^ ^66^, ^67^ ^]^ Interestingly, artificial secretory granules show also structural dynamism in vitro, in absence of any exogenous protein‐based folding modulator (Figure 3C). In fact, Zn itself is a folding modulator, inducing conformational changes in a cooperative manner when interacting with various proteins or peptides.^[^ ^68^, ^69^, ^70^, ^71^ ^]^

In this context, no structural stabilization has been observed in T22‐GFP‐H6 when using Ca^2+^ or Mn^2+^ as clustering agents (Figure 3). This is interestingly associated with lower protein leakage from materials based on Ca^2+^ and Mn^2+^, compared to materials based on Zn^2+^, under identical conditions (Figure 2A). Such coincidence might be indicative of that the stabilization of the oligomeric status promoted by Zn ^2+^ might positively influence the capability of the protein to detach from the granules under suitable conditions. It could be not discarded that the conformational fluctuations induced by Zn ^2+ [^ ^72^ ^]^ might increase the motility of the protein within the granule making it more suitable for release, in contrast with granules generated through Ca ^2+^ and Mn ^2+^, which are not so closely linked to dynamic protein folding/misfolding events.

## 4 Conclusion

The controlled coordination of Zn^2+^ and H6‐tagged proteins generates submicron particles or MPs that slowly release the forming polypeptide in a functional form, like natural amyloidal secretory granules. These materials show promise in clinical settings for which a time‐prolonged delivery from protein drug depots is required. The data presented in this study, using several unassembled and self‐assembling proteins as convenient models, demonstrate that the aggregation and the consequent release events do not correspond to mere attachment/detachment processes of the building blocks, but rather to an intimate protein integration into the generated material, during its generation, which compromises its conformational status. The structural complexity of one paradigmatic model, the homomeric T22‐GFP‐H6 nanoparticles whose size largely depends on the protein conformation, has provided a highly sensitive instrument that magnifies those structural changes, allowing their quantitative visualization. Such integration of oligomeric building blocks into the material results into a structural stabilization that make the released nanoparticles slightly larger and more resistant to thermal stress than the initial soluble versions. On the other hand, the conformation‐adapted material versions are released from MPs at higher efficiency than the equivalents that keep the original conformation (Figure 3C). In practical terms and as demonstrated here, microscale protein granules fabricated in vitro through Zn/His coordination represent endocrine‐like depots that are extremely promising instruments for in vivo drug delivery, aiming at the steady supply of functional protein (Figure 5). Being these facts deeply linked to Zn as a clustering agent for MP fabrication, the whole set of data allows discarding alternative divalent cations for protein clustering into secretory depots, while it also accounts for the generic presence of ionic Zn in amyloidal natural peptide depots with secretory properties.

## 5 Experimental Section

### Protein Material

T22‐GFP‐H6 is a fusion protein with modular architecture that upon production in bacteria self assembles as regular oligomeric nanoparticles ^[^ ^17^ ^]^ of ≈10 nm. Both electrostatic forces and coordination of the C‐terminal H6 tail with metallic cations from the media promote and stabilize, respectively, the protein complexes.^[^ ^73^ ^]^ Because of the N‐terminal peptide T22, a polyphemusin peptide analogue that precisely binds the tumoral marker CXCR4,^[^ ^74^, ^75^, ^76^, ^77^, ^78^ ^]^ T22‐GFP‐H6 nanoparticles selectively bind and internalize CXCR4‐overexpressing metastatic cancer stem cells, being suited as drug carriers for precision oncological treatments.^[^ ^11^ ^]^ Production and purification of T22‐GFP‐H6 were performed by conventional procedures as described.^[^ ^11^ ^]^ Other fusion proteins was incorporated for comparative analyses, namely GFP‐H6,^[^ ^16^ ^]^ β‐Gal‐H6 ^[^ ^79^ ^]^ and EPIX4‐RK‐GFP‐H6,^[^ ^15^ ^]^ whose production and purification procedures were previously documented.^[^ ^15^, ^16^, ^79^ ^]^ The storage solution for each protein was carbonate with salt (166 mM NaCO_3_H, 333 mM NaCl, pH 8) for T22‐GFP‐H6; carbonate (166 mM NaCO_3_H, pH 8) for EPIX‐RK‐GFP‐H6; PBS 1x, pH 7.4 for β‐Gal‐H6 and phosphate buffer, pH 7.4 for GFP‐H6. These buffers were used during the characterization of each respective protein.

### Preparation of Microscale Secretory Granules

Aliquots of 2 mg mL^−1^ T22‐GFP‐H6 were gently mixed and incubated at room temperature for 10 min, with salt solutions to reach the following final concentrations: 10 mM ZnCl_2_, 100 mM CaCl_2_, 50 mM MnCl_2_, or 6 mM ZnCl_2_ and 54 mM CaCl_2_ mM. For other protein species, 2 mg mL^−1^ of protein was mixed with 10 mM ZnCl_2_ for the formation of MPs. These precise salt concentrations were selected to achieve a complete precipitation of the protein into insoluble MPs as described,^[^ ^41^ ^]^ by coordination between the divalent cations and the H6 tail. The clustered materials were recovered by sedimentation upon centrifugation of the mixtures at 10 000 g, for 5 min, at room temperature. A more precise description of the procedure can be found elsewhere.^[^ ^41^ ^]^

### Analysis of Protein Release from Secretory Granules

Selected MPs, in 500 µL of storage solution and at 1 mg mL^−1^ were incubated at 37 °C for 7 days without agitation. The solutions were carbonate with salt (166 mM NaCO_3_H, 333 mM NaCl, pH 8) for T22‐GFP‐H6, carbonate (166 mM NaCO_3_H, pH 8) for EPIX‐RK‐GFP‐H6, PBS 1x, pH 7.4 for β‐Gal‐H6 and phosphate, pH 7.4 for GFP‐H6. After 7 days, samples were centrifuged at 15 000 g, for 15 min, at 4 °C, and supernatants (*OUT* samples) were separated from the pellet for further analysis. The amount of released protein was determined upon SDS‐PAGE and further band densitometry analysis in a TGX stain‐free gel (Bio‐Rad). Protein bands were quantified with Image lab. Protein bands were also detected by Western Blot by transferring with a Trans‐Blot Turbo Transfer System (Bio‐Rad) into polyvinylidene fluoride (PVDF)membranes and immunodetecting with an anti‐GFP (Santa Cruz, Biotechnology) mouse monoclonal antibody. A conventional Bradford assay was employed for the quantitative analysis of the released protein and these values used to correct the circular dichroism and fluorescence spectral raw data to allow comparative analyses. Two additional protein samples or *IN* samples were included in the study. These were a control sample that consisted of 500 µL of T22‐GFP‐H6 at 1 mg mL^−1^ in carbonate solution incubated at 4 °C for 7 days, and the so‐called 37 °C sample that contained 500 µL of T22‐GFP‐H6 at 1 mg mL^−1^ in carbonate solution, incubated at 37 °C for 7 days.

### Electron Microscopy

High‐resolution images of T22‐GFP‐H6 nanoparticles and granules were obtained by TEM. For that, 10 µL droplets of protein (at 0.02 mg mL^−1^) were placed on top of glow‐discharged 200 mesh carbon‐coated copper grids (Electron Microscopy Sciences) for 1 min. The excess of liquid was blotted with Whatman filter paper. Then, negative staining was performed by placing the grids on top of a 10 µL drop of 1% uranyl acetate (Polysciences Inc.) for 30 s and the excess liquid blotted again. Grids were dried at room temperature for at least 10 min and the images were acquired in a JEOL JEM‐1400 (Jeol Ltd.) transmission electron microscope operating at 80 kV and equipped with a Gatan Orius 8 9 SC200 CCD camera (Gatan Inc.). Representative images were captured from different fields at 20 000x magnification.

### Analysis of Protein Secondary Structure

The secondary structure of *IN* and of the released‐*OUT* protein was analysed by circular dichroism (CD). Spectra were acquired with a spectropolarimeter JASCO J‐715 (JASCO, Oklahoma City, OK) applying a 0.2 mm path length quartz cell. Each spectrum was an average of seven scans. Scan speed was set at 100 nm min^−1^ with a 1 s response time. Measurements were obtained as ellipticity (θ) in millidegrees (mdeg) in the of 200–260 nm range. The calculation of θ_216_/θ_210_ ratio named as secondary structure parameter (SSP) from each spectrum allowed us to compare the CD spectrum movement respect to that of the control protein. Data are expressed as mean ± standard error. To assess the secondary structure content of GFP‐H6 was also determined in the range of 180–260 and the further analysis was performed with Dichroweb (^[^ ^80^ ^]^ and references therein) using the self‐consistent method.

### Analysis of Protein Tertiary Structure

For tertiary structure analysis, the GFP fluorescence emission spectra in a Cary Eclipse spectrofluorometer (Agilent Technologies) with a quartz cell of 2 mm path length was determined. The excitation slit was set at 2.5 nm and emission slit at 5 nm. The excitation wavelength (λ_ex_) was 488 nm. The fluorescence emission spectra were acquired within a range from 500 to 600 nm. The calculation of IF_513_/IF_508_ ratio from each spectrum allowed the comparison of the fluorescence spectrum change regarding the control protein. Data are expressed as mean ± standard error. The same equipment was applied to study the T22‐GFP‐H6 and β‐Gal‐H6 intrinsic fluorescence but in this case, the excitation and emission slits were set at 5 nm. Excitation wavelength (λex) was set at 295 nm. Emission spectra were acquired within a range from 310 to 450 nm. The protein concentration was 0.2 mg mL^−1^ in the corresponding buffer. The fluorescence signal of T22‐GFP‐H6 mainly belongs to the Trp located in the T22 peptide. On the other side, β‐Gal‐H6 contains 156 Trp residues distributed across the protein. Therefore, a possible change in compactness/expansion of the *OUT* protein respect to the *IN* version can be assumed. For the analysis, we applied the Centre of Spectral Mass (CSM). CSM is a weighted average of the fluorescence spectrum peak, and it is related with the relative exposure of the Trp to the protein environment. The maximum redshift in the CSM of the Trp, is compatible with a large solvent accessibility. The Centre of Spectral Mass (CSM) was calculated as described.^[^ ^14^ ^]^

### Analysis of the Oligomeric State

The size of the assembled proteins was determined by dynamic light scattering (DLS) at 633 nm and 25 °C in a Zetasizer Advance Pro (Malvern Instruments), in triplicate. For disassembling, *IN*, MPs and *OUT* proteins were treated with 30, 60 and 30 mM of EDTA (a chelating agent) respectively for 5 mins.^[^ ^16^ ^]^ Data are expressed as mean ± standard error. The protomeric configuration of the protein released from Mn‐MPs was assessed by blue native gel electrophoresis. Native protein mixed with loading buffer were loaded in an 8% polyacrylamide gel. Running conditions were 20 mA in TAE 1x buffer at 20 °C.

### Amyloid Content in Microparticles

The presence of cross β‐structure was determined by Fourier transform infrared spectroscopy coupled to an attenuated total reflectance (ATR‐FTIR). Protein MPs were placed on a spectroscopic crystal surface. Measurements were performed 16 times in a continuous flow of nitrogen. Spectra were recorded at a scan rate of 50 cm^−1^ min^−1^ with a nominal resolution of 2 cm^−1^, in a Tensor 27 Bruker spectrometer with a Specac's Golden Gate Attenuated Total Reflectance (ATR) accessory. The absorbance values were corrected by subtracting the background. Fourier deconvolution of the spectra and the second derivative allowed the identification and analyses of different band components. Data were treated by using the Peakfit software.^[^ ^5^ ^]^ In addition, we applied the Thioflavin T assay to evaluate the presence of amyloidal structure within β‐Gal‐H6 MPs as in this case, there was no interference with a fluorescence protein. To a β‐Gal‐H6 sample at 0.1 mg mL^−1^ Thio T (Sigma–Aldrich) in PBS was added to 25 µM. ThioT fluorescence was excited at 450 nm, and the fluorescence emission spectra were recorded with a Varian Cary Eclipse spectrofluorometer in the 470−600 nm range. The cross‐β‐sheet structure of the β‐Gal‐H6 MPs was monitored by the increase of the free dye fluorescence emission caused by the interaction with the amyloidogenic protein.

### Animal Manipulation and Data Analysis

Experiments were conducted following the European Council directives and approved by the Animal Ethics Committee at Hospital de la Santa Creu i Sant (procedure 9721). Female Swiss nude mice, aged 6–8 weeks, and weighing between 16–22 g, were obtained from Charles River (L'Arbresle, France), and were housed in a sterile environment with inbedding, water, and γ‐ray‐sterilized food ad libitum. The M5 patient‐derived colorectal cancer line was established from a sample of the primary tumor resected from a colorectal cancer (CRC) resistant to chemotherapy from a patient at the Hospital de Sant Pau in Barcelona. This line was used to generate the subcutaneous (SC) cancer model in immunosuppressed mice. This tumor was classified as a large and poorly differentiated adenocarcinoma, and displayed high CXCR4 expression. It was first implanted donor mice with the patient‐derived M5 line. When M5 tumor volume in donor mice reached ≈400 mm^3^, tumors were excised and reimplanted in mice to generate the experimental SC CRC model. Once tumors reached ≈120 mm^3^, mice were randomized in three groups: control solution, soluble T22‐GFP‐H6 *IN* material and T22‐GFP‐H6 MPs formed by Zn‐mediated clustering, which were subcutaneously injected to the corresponding mouse. Following, we measured the fluorescence accumulation in the remote tumor tissues at day 1 and at 10. This would allow comparing the protein released pattern between the *IN* soluble protein and the MPs. Thus, a single dose of 1000 µg of MPs, or one dose of 1000 µg of *IN* soluble protein was administered by subcutaneous route, in the mouse lumbar side opposite to that of the tumor, using a pellet of protein microgranules suspended in 50 µL of carbonate solution or 50 µL of *IN* soluble protein. The monitoring of the protein uptake into the tumor at day 1 and day 10 of the study was carried out after de necropsy by *ex vivo* recording of the tumor GFP fluorescence in the IVIS spectrum at the both studied times. The Mann–Whitney test was used to compare tumor tissue fluorescence emission (FLI) between groups using GraphPad Prims 8.0.2. Differences between groups were considered significant at p < 0.05.

## Conflict of Interest

The authors declare no conflict of interest.

## Supporting information

Supporting Information

## Data Availability

The data that support the findings of this study are available from the corresponding author upon reasonable request.
