# Supplementary material for: Structural Stabilization of Clinically Oriented Oligomeric Proteins During their Transit through Synthetic Secretory Amyloids
Source: Adv Sci (Weinh). 2024 Mar 19;11(21):2309427. doi: 10.1002/advs.202309427 (PMC11151067; doi:10.1002/advs.202309427)
Supplement: Supplementary file 1 — Supporting Information [file ADVS-11-2309427-s001.pdf]

ADVANCED SCIENCE
Open Access

Supporting Information

for *Adv. Sci.*, DOI 10.1002/advs.202309427

Structural Stabilization of Clinically Oriented Oligomeric Proteins During their Transit through Synthetic Secretory Amyloids

Julieta M. Sánchez, Hèctor López-Laguna, Eloi Parladé, Angela Di Somma, Andrea L. Livieri, Patricia Álamo, Ramón Mangues, Ugutz Unzueta, Antonio Villaverde and Esther Vázquez**

Supplementary materials

Structural Stabilization of Clinically Oriented Oligomeric Proteins During their Transit through Synthetic Secretory Amyloids

Julieta M. Sánchez, Hèctor López-Laguna, Eloi Parladé, Angela Di Somma, Andrea Livieri, Patricia Álamo, Ramón Mangues, Ugutz Unzueta, Antonio Villaverde, Esther Vázquez**

Julieta M. Sánchez, Hèctor López-Laguna, Eloi Parladé, Angela Di Somma, Andrea Livieri, Antonio Villaverde, Esther Vázquez
Institut de Biotecnologia i de Biomedicina, Universitat Autònoma de Barcelona, Plaça Cívica s/n, Bellaterra, 08193 Barcelona, Spain
Julieta M. Sánchez, Héctor López-Laguna, Antonio Villaverde, Esther Vázquez
Departament de Genètica i de Microbiologia, Universitat Autònoma de Barcelona, Plaça Cívica s/n, Bellaterra, 08193 Barcelona, Spain
Julieta M. Sánchez, Héctor López-Laguna, Eloi Parladé, Ugutz Unzueta, Antonio Villaverde, Esther Vázquez
CIBER de Bioingeniería, Biomateriales y Nanomedicina (CIBER-BBN), C/ Monforte de Lemos 3-5, 28029 Madrid, Spain
Julieta M. Sánchez
Instituto de Investigaciones Biológicas y Tecnológicas (IIBYT) (CONICET-Universidad Nacional de Córdoba), ICTA, FCEFyN, UNC. Av. Velez Sarsfield 1611, Córdoba X 5016GCA, Argentina
Patricia Álamo, Ramón Mangues, Ugutz Unzueta
Institut d'Investigació Biomèdica Sant Pau (IIB SANT PAU), Sant Quintí 77-79, 08041 Barcelona, Spain
Patricia Álamo, Ramón Mangues, Ugutz Unzueta
Josep Carreras Leukaemia Research Institute, 08025 Barcelona, Spain.
Angela Di Somma
Department of Chemical Sciences, University of Naples "Federico II", Vicinale Cupa Cintia 26, 20126 Naples, Italy
Angela Di Somma
CEINGE Advanced Biotechnologies, Via Gaetano Salvatore 486, 80131 Naples, Italy

E-mail: EV, Esther.Vazquez@uab.es; AV, Antoni.villaverde@uab.es

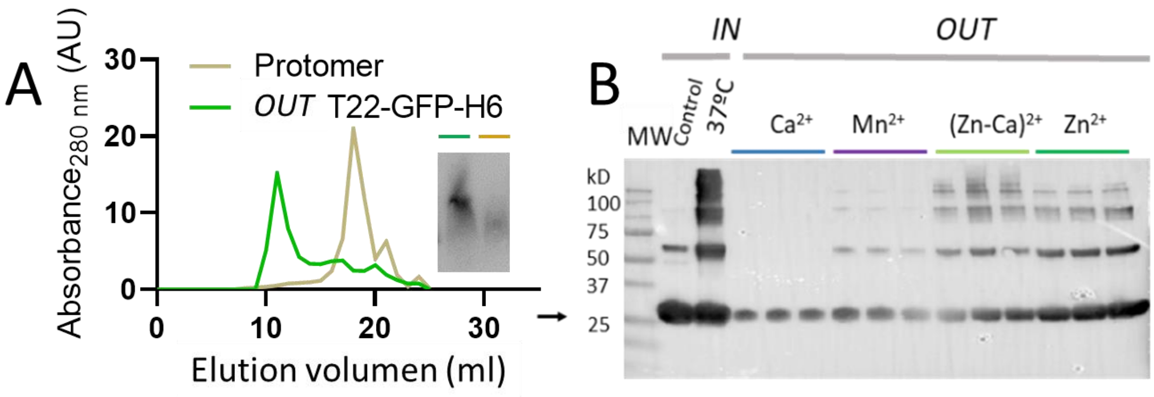

Figure S1. A. Size exclusion chromatography (SEC) of *OUT* protein and the T22-GFP-H6 protomer. In the inset, native PAGE of both samples. **B. Protein release from secretory granules.** Western blot of *OUT* T22-GFP-H6 (arrow) released from MPs formed by different clustering cations, corresponding to the samples analysed by TGX and shown in the Figure 2A. Samples were taken upon in vitro incubation of freshly prepared granules at 37ºC, in buffer, for 7 days, and processed in triplicate. Control *IN* protein either stored for 7 days at 37ºC or kept at 4ºC (Control) are also shown as references. A dimeric form of the protein appears as a band slightly above of that of 50 kDa.

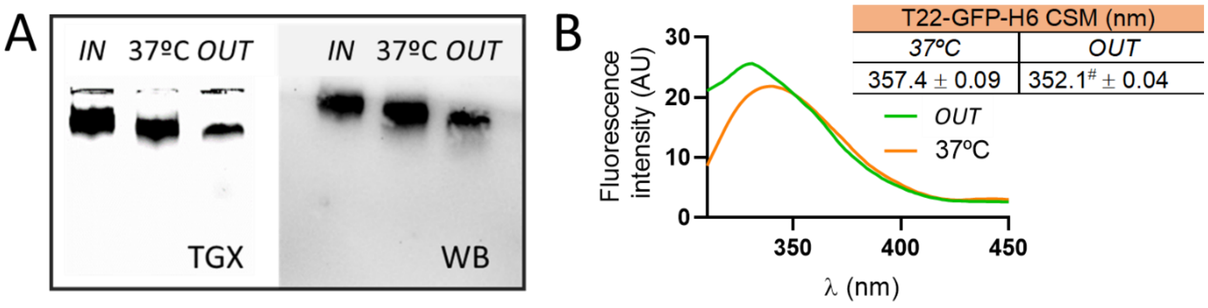

Figure S2 A. Native gel electrophoresis, showing TGX and Western Blot (WB) visualizations. Samples: *IN*: Soluble T22-GFP-H6 kept at 4ºC, 37ºC: Soluble T22-GFP-H6 incubated at 37ºC for 7 days and OUT: T22-GFP-H6 released from Mn-MPs. **B. Fluorescence emission of tryptophan.** It was determined from *OUT* T22-GFP-H6 releaseed from Zn-MPs and the soluble form kept at 37ºC for 7 days (λex=295 nm). The table shows the center of spectral mass values, calculated as described in the experimental section (**#,** statistically different p<0.001).

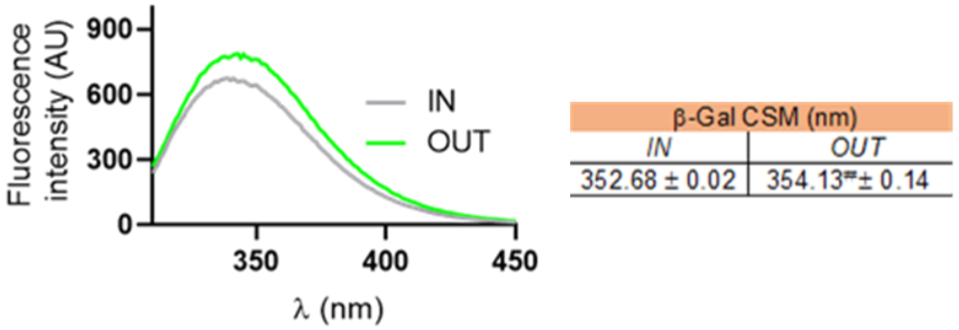

Figure S3. Fluorescence emission of tryptophan from *IN* and *OUT* β-Gal-H6 (λ_{ex}=295 nm). The table shows the center of spectral mass values, calculated as described in the methods section. (# Statistically different p<0.001).

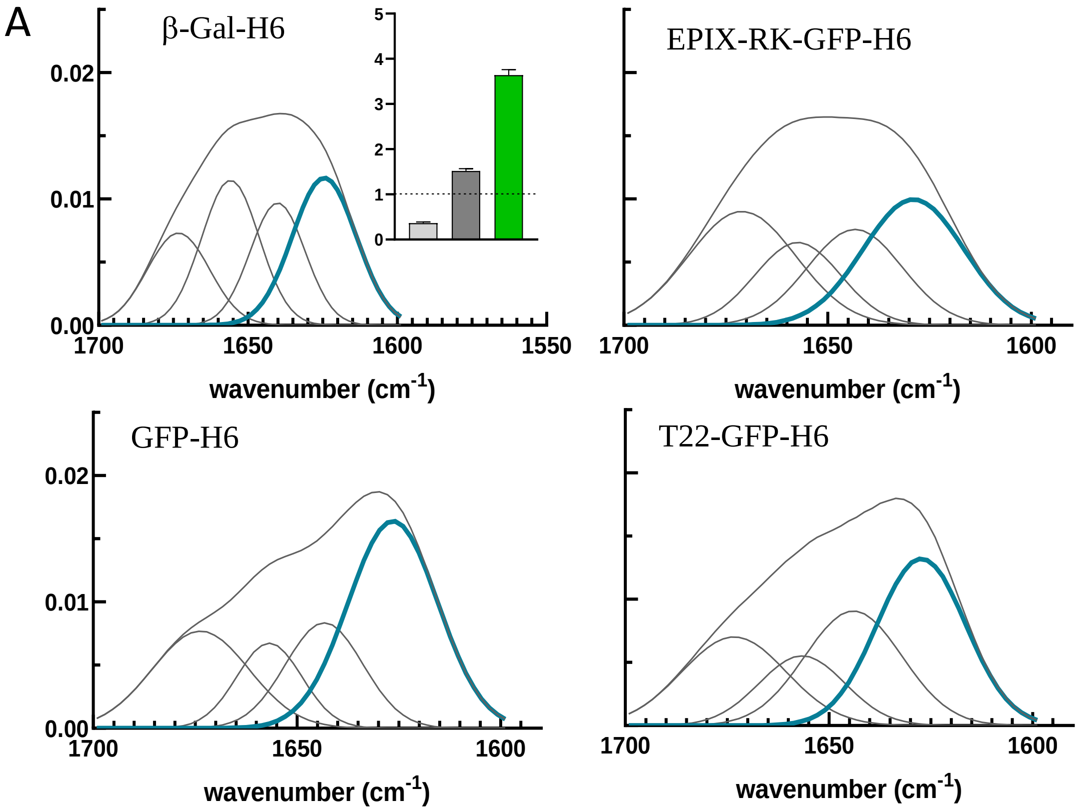

B

Peak Position (cm^{-1})	Component	Structure (%)			
		**β-Gal-H6**	**EPIX-RK-GFP-H6**	**GFP-H6**	**T22-GFP-H6**
1622-1627	Amyloid-like	31.3 ± 1.1	33.3 ± 2.6	44.4 ± 3.7	39.4 ± 3.2
1644-1648	Random coil	22.7 ± 3	23.7 ± 1.6	19.6 ± 0.4	25.9 ± 2.6
1655-1665	Alpha helix	28.5 ± 1.9	16.8 ± 1.6	13.9 ± 0.6	14.4 ± 1.3
1671-1679	High. Frec. Antipar. β-sheet	19.9 ± 1.6	25.9 ± 3.5	24.7 ± 1.9	22.2 ± 1.1

Figure S4. Amyloid content analysis. A ATR-FTIR spectra of MPs formed by β-Gal-H6, EPIX-RK-GFP-H6, GFP-H6 and T22-GFP-H6. The amyloid structure is indicated in blue after the deconvolution analysis for each MP dataset. In the inset, the amyloidal structure of β-Gal-H6 determined by Thioflavin T (Thio T). Thio T control (dashed line), soluble β-Gal-H6 (dark grey bar), $ZnCl_2$ (light grey bar) and β-Gal-H6 MPs (green bar). **B.** The percentage (%) of each type of secondary structure is shown, as determined from the peak fit analysis. The amyloid structure is shown in the yellow file.

Table S1. Secondary structure analysis of GFP-H6 by applying the software Dichroweb on the circular dichroism spectra of *IN* and *OUT* samples and of the *IN* protein sample kept at 37ºC.

**Protein condition**	Structural rate			
	**Alpha helix**	**Beta structure**	**Turns**	**Unordered structure**
IN	0.003± 0.041	0.553 ± 0.082	0.188 ± 0.0255	0.208 ± 0.101
37ºC	-0.006± 0.053	0.563 ± 0.0838	0.171 ± 0.0722	0.281 ± 0.0350
OUT	-0.031± 0.0583	0.575 ± 0.0597	0.204 ± 0.0768	0.275 ± 0.0399

*non statistically difference between each structure rate of protein samples. (NRMSD=0.38)
